# Evidence of extrinsic factors dominating intrinsic blood host preferences of major African malaria vectors

**DOI:** 10.1038/s41598-020-57732-1

**Published:** 2020-01-20

**Authors:** James Orsborne, Abdul Rahim Mohammed, Claire L. Jeffries, Mojca Kristan, Yaw A. Afrane, Thomas Walker, Laith Yakob

**Affiliations:** 1 Department of Disease Control, London School of Hygiene & Tropical Medicine, London, UK; 2 Department of Medical Microbiology, College of Health Sciences, University of Ghana, Korle Bu, Accra Ghana

**Keywords:** Entomology, Malaria

## Abstract

One of the key determinants of a haematophagous vector’s capacity to transmit pathogens is its selection of which host to secure a blood meal from. This choice is influenced by both intrinsic (genetic) and extrinsic (environmental) factors, but little is known of their relative contributions. Blood fed *Anopheles* mosquitoes were collected from a malaria endemic village in Ghana. Collections were conducted across a range of different host availabilities and from both indoor and outdoor locations. These environmental factors were shown to impact dramatically the host choice of caught malaria vectors: mosquitoes caught indoors were ten-fold more likely to have sourced their blood meal from humans; and a halving in odds of being human-fed was found for mosquitoes caught only 25 m from the centre of the village. For the first time, we demonstrate that anthropophagy was better explained by extrinsic factors (namely, local host availability and indoor/outdoor trapping location) than intrinsic factors (namely, the (sibling) species of the mosquito caught) (respective Akaike information criterion estimates: 243.0 versus 359.8). Instead of characterizing biting behaviour on a taxonomic level, we illustrate the importance of assessing local entomology. Accounting for this behavioural plasticity is important, both in terms of measuring effectiveness of control programmes and in informing optimal disease control strategies.

## Introduction

Mosquitoes are responsible for the transmission of multiple human pathogens with malaria the most prominent of the resulting diseases. Malaria is transmitted by the bite of several *Anopheles* mosquito species when infected with *Plasmodium* parasites. As the successful transmission of the parasite requires two successful bites (one for the mosquito to become infected and one for the parasite to be transmitted to the human host), the mosquito’s choice of which host species to bite has an exaggerated, non-linear impact on malaria transmission intensity^1^. The way in which host availability impacts the choice of host is largely unknown despite the dramatic impact it has on transmission and control of vector-borne diseases of diverse aetiology^2^. Similarly little is known of the short- and long-term impacts of different control methods on host choice.

The introduction of indoor residual spraying and insecticidal nets has resulted in a change in the biting behaviour of major malaria vectors with many mosquito species seeking a higher proportion of blood meals outdoors^3–6^ and/or from non-human hosts^7–10^. Coupled with increasing rates of insecticide resistance^11^, these behavioural changes indicate potential inadequacies of current leading vector-control tools to target outdoor biting and, correspondingly, sustained residual malaria transmission^12–19^. Therefore, understanding this behaviour is becoming increasingly important when implementing vector control strategies.

In the field, which host is ultimately bitten is a complex balance between intrinsic (genetic) and extrinsic (environmental) factors including behavioural conditioning driven by exposure to insecticides or previous successful feeds from a particular host^20,21^. The amalgamation and balance of these factors results in a significant level of host biting plasticty which can have considerable consequences for disease control^2^. One of the most popular methods of investigating host biting behaviour in the field is to collect blood fed mosquitoes and identify the blood meal source. The proportion (or percentage) of blood meals that are of human origin is referred to as the Human Blood Index (HBI)^22^. Malaria-transmitting mosquitoes are often described as primary or secondary vectors with this categorisation informed, in part, by their level of anthropophagy as indicated by their HBI. Owing to the influences of different environmental settings, however, studies reporting HBI for the same vector species demonstrate considerable variability^21,23–25^. We recently showed that the HBI of *Anopheles coluzzii* varied significantly over an extraordinarily small spatial scale, thus demonstrating how localised host biting behaviour can be^26^. Further, a recent systematic review indicated that the location a mosquito was caught (indoors versus outdoors) may have just as much influence, if not more influence, on host selection than the taxa collected^23^. However, these studies were performed by multiple researchers using a variety of methods on different mosquito populations, and spanned several decades. To the best of our knowledge, the relative influence of intrinsic and extrinsic factors on host selection has not been reported for any major disease vector.

In this study, multiple species of blood fed *Anopheles* mosquitoes were collected across a range of host availabilities, both from indoors and outdoors. Our hypothesis was that local environmental factors had a greater influence over blood host selection than the (sibling) species of the caught mosquitoes. We discuss the consequences of our findings to assessing, and perhaps even augmenting, future control strategies.

## Methods

### Study site and mosquito collection

**Figure 1:**
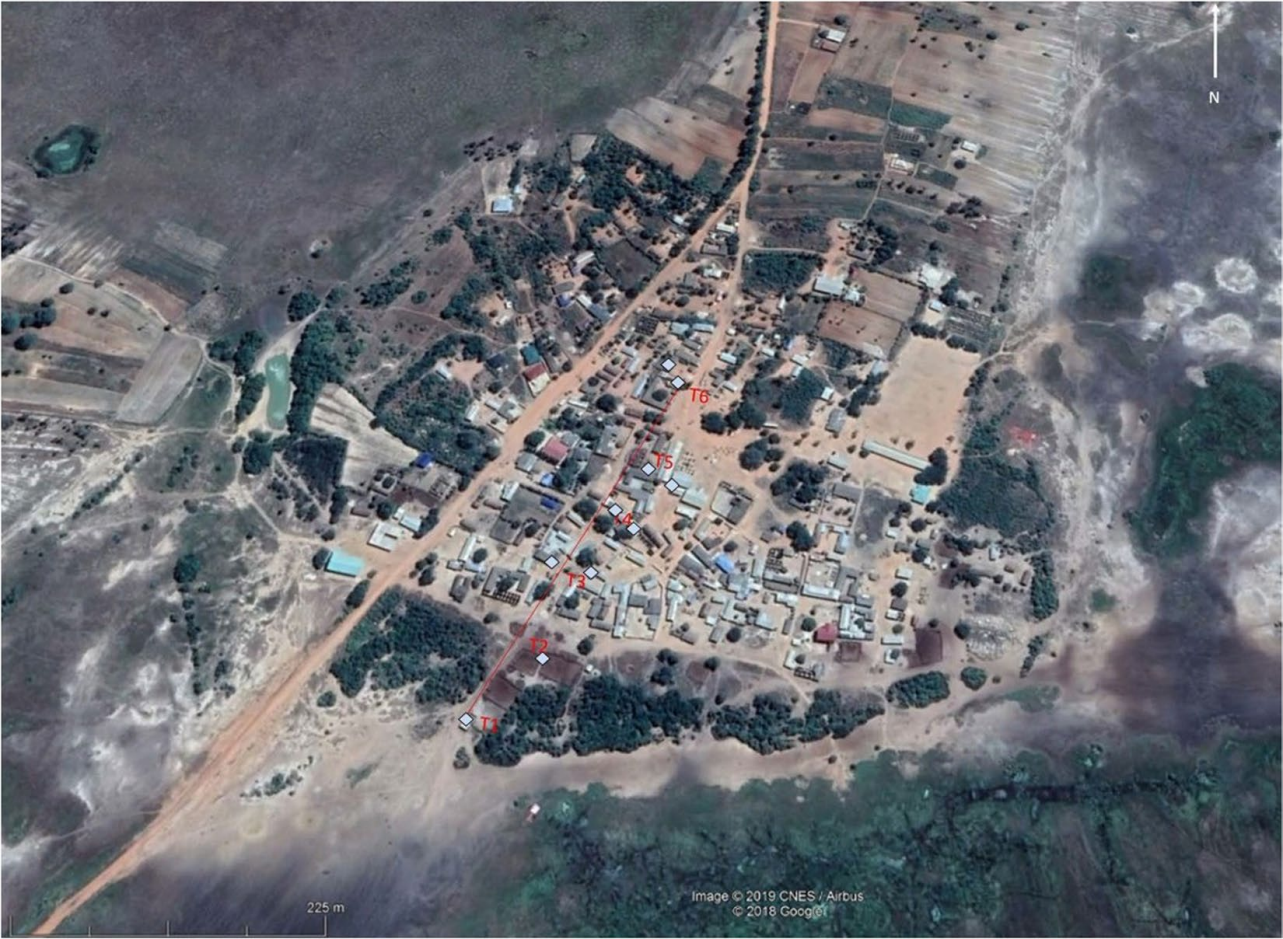
Map of collection site and transect points (shown in red). Location of buildings and houses used for indoor sampling for each transect point are indicated by the blue squares.

Mosquitoes were collected from the village of Dogo, in the Greater Accra region of Ghana (05°52.418N, 00°33.607E) over 22 nights across June and July 2018 (Fig. 1). The village is on the south-eastern coast of Ghana, with a main rainy season from April to June and a shorter second rainy season in October. Temperatures were measured using a datalogger (EasyLog USB, UK) with windspeed recorded using an anemometer (Holdpeak, UK). Housing mostly consisted of concrete structures with concrete/brick walls and flooring, with predominantly iron roofs although some traditional mud style houses were also present. Several criteria were used to select the site: it had a defined area where cattle were kept and rested during the collection period on the outskirts of the village; the village had no other nearby cattle holdings; human housing density gradually increased towards the village centre and away from the cattle pen. Critical for this site being selected was that it offered both indoor and outdoor mosquito collection points within a full range of availabilities of alternative host species. Mosquito species known to be present in the area were primarily *An. gambiae* and *An. coluzzii* as well as *An.pharoensis* and *An. rufipes*. Due to the proximity to the sea, the potenital of *An. melas* being present was also noted and considered during molecular analysis.

Outdoor mosquito collections consisted of three outdoor trapping types: Centers for Disease Control and Prevention (CDC) resting traps (Bioquip products, CA, USA), BG Sentinel 2 traps (Biogents AG, Regensburg, Germany) and CDC light traps (Bioquip products, CA, USA). These traps were placed in clusters (<10 m apart) at 50 m intervals to form a 250 m transect comprising of six trapping points (Fig. 1). The transect began at an area of zero human population density (termed ‘T1’) outside of Dogo village and nearby the cattle resting and overnight holding pen. The transect extended towards a human population ending at an area of high human density, the middle of Dogo village (denoted ‘T6’). Details of hosts located at each transect point are described in Table 1. Mosquitoes were collected overnight from 6 pm to 6 am. Indoor collections were performed using a Prokopack aspirator (John.W.Hock, FL, USA) at two houses per transect point within the village (T3–T6) with each house being alternated for each collection night. Indoor collections at T1 and T2 were performed in outbuildings, which had no residents. Indoor collections were performed for 15 minutes in each building between 4 am and 6 am of each collection night. Bednets were absent from most households and only seen to be used sporadically in remaining households during sampling.

**Table 1:** Approximate number of hosts across the transect.

Transect	Approximate number of hosts in area	Approximate number of indoor hosts
1	cows n = 150	n = 0, empty house used for sorting farming and cattle rearing equipment, open window frames and eves
2	cows n = 150 pigs n = 5	n = 0, empty cattle shed with open window frames but door closed to stop animals resting within
3	humans n = approx.’ 20 chickens n = 7 dogs n = 3 goats n = 4	n = 6 permanent human inhabitants, dogs sometimes found in porch at night
4	humans n = approx.’ 30 guinea fowl n = 3 cats n = 2	n = 7 permanent human inhabitants
5	humans n = approx.’ 45	n = 6 permanent human inhabitants
6	humans n = approx.’ 85 chickens n = 3	n = 9 permanent human inhabitants

### Ethical clearance

The study was reviewed and cleared by the London School of Hygiene & Tropical Medicine (LSHTM) ethics committee (LSHTM ethics reference: 15216). Ethical clearance was also obtained in country and cleared by the University of Ghana (UOG) ethics committee (University of Ghana, Noguchi Memorial Institute reference: DF22). Ethical clearance was obtained prior to any part of the study being performed. All methods in this study were performed in accordance with all relevant guidelines and regulations.

### Recruitment of households for indoor collection

Households within 10 metres of each outdoor trap cluster were approached to be recruited into the study. Households were provided with a participant information sheet explaining the study and for those who could not read or speak English, a local translator was present. If the head of the household agreed to take part in the study, informed consent was provided via the signature of a consent form and the household was recruited into the study. All households were allowed to withdraw at any time during the study.

### Morphological identification of mosquito species

*Anopheles pharoensis* and *An. rufipes* are morphologically distinguishable from the *An. gambiae* complex as well as from each other. Morphological Identification of these species was performed in the field prior to sample processing using a key developed by Gillies and Coetzee^27^. *An*. *gambiae* were identified only to the species complex until molecular analysis.

### Sella staging of blood-fed mosquitoes

The Sella score is a visual measure of blood meal digestion and is comprised of seven stages. Stage two (II) is a freshly blood fed mosquito and stage seven (VII) is a fully gravid mosquito, stages between are defined by set criteria. Each blood fed mosquito in this study was assessed under light microscope and scored using the Sella score originally described by Detinova^28^.

### Sample storage

*Anopheles* mosquitoes collected from each transect point were immediately killed using chloroform to stop any active blood meal digestion. Mosquitoes where then sorted with all blood-fed females being processed first. All overtly blood-fed *Anopheles* mosquitoes were processed individually, noting their Sella score^28^ (a visual measure of blood meal digestion), transect location, trap type, night collected and collection location (indoor or outdoor). All blood-fed mosquitoes were preserved in RNAlater® (Thermo Fisher Scientific, Life Technologies,UK) in a 96 well plate and stored at 4 °C and transported in this format back to the UK. All samples were stored at −70 °C once transported back to laboratories at the London School of Hygiene & Tropical Medicine.

### DNA extraction

DNA was extracted from each *Anopheles* mosquito individually. Each sample was firstly homogenised using a Qiagen TissueLyser II (Qiagen, UK) with a 5 mm stainless steel bead (Qiagen, UK) and placed in each sample tube in a 96 well plate format (Qiagen UK). Once homogenised, DNA was extracted using the Qiagen DNeasy 96 kits (Qiagen, UK) following the manufacturer’s protocol. Extracted DNA was stored at −20 °C until analysis was performed.

### *An. gambiae* species complex identification

Mosquito species identification first involved a real-time multiplex PCR assay targeting the rRNA gene developed by Bass *et al*.^29^. Standard forward and reverse primers were used in conjunction with two species-specific Taqman© probes. The reaction conditions were as follows: a 12.5 µl reaction containing 1 µl of genomic DNA. 6.25 µl of QuantiNova (Qiagen, UK) probe master mix and 2.9 µl of DNA free H_2_O. 800 nM of forward and reverse primers (Thermo Fischer Scientific, UK), 200 nM of *An. arabiensis* probe (Sigma-Aldrich, UK) and 80 nM of *An. gambiae* probe (Applied Biosystems, UK). Samples were run on a Stratagene MX3005P (Agilent Technologies, USA) using cycling conditions of 10 min at 95 °C, followed by 40 cycles of 95 °C for 25 s and 66 °C for 60 s. The increases in fluorescence were monitored in real time by acquiring at the end of each cycle. Analysis was carried out using the Stratagene MxPro software.

### *An. coluzzii* and *An. gambiae* sensu stricto identification

To differentiate between *An. coluzzii* and *An. gambiae* s.s. within the *An. gambiae* species complex, a single end-point PCR was performed. This PCR targets the SINE200 retrotransposon and utilising an insertion in this area allows the two species to be distinguished following gel visualisation^30^. *An. coluzzii* produces a band at 479 bp with *An. gambiae* producing a band at 249 bp. Reaction was as follows: a 25 µl reaction containing 0.5 mM of forward and reverse primers, 12.5 µl of Hot start Taq polymerase (New England Biolabs NEB, UK), 9.5 µl of nuclease-free water and 2 µl of template DNA. Cycling conditions were as follows: 10 min at 94 °C followed by 35 cycles of 94 °C for 30 s, 54 °C for 30 s, 72 °C for 60 s, a final elongation step of 72 °C for 10 minutes finished the cycling program.

### *An. melas* identification

*An. melas* has been found in coastal areas of Ghana. With the study site close to the sea and <5 km from saltwater lagoons, an endpoint assay developed by Scott *et al*.^31^ was used to identify this *An. gambiae* sibling species. The PCR targets the ribosomal rDNA gene and used a universal forward primer with species-specific reverse primers to produce different product sizes allowing species to be identified. The product size was 464 bp for *An. melas*. Reaction volume consisted of 10 µl of Hot start Taq polymerase (New England Biolabs NEB, UK), 2 µl of universal forward primer (10 µM) 2 µl of *An. melas* specific reverse primer (10 µM), 4 µl of DNA/RNA free H_2_O and 2 µl of template DNA. Cycling conditions were as follows: 10 mins at 95 °C followed by 30 cycles of 30 s at 95 °C, 30 s at 50 °C and 30 s at 72 °C and a final elongation stage of 5 mins at 72 °C.

### End-point PCR gel visulisation

All endpoint PCR products were visualised on a 2% agarose gel cartridge using an Egel E-Gel iBase Power System and E-Gel Safe Imager Real-Time Transilluminator (Invitrogen, UK).

### Blood meal identification

Due to the nature of the experimental set up with humans and bovines dominating all blood meal sources, samples were initially screened using bovine and human specific primers developed by Gunathilaka *et al*.^32^. The reaction conditions consisted of a 10 µl reaction including 0.5 M of forward and reverse primers (Integrated DNA Technologies), 5 µl of SYBR green master mix (Roche, UK), 2 µl of nuclease-free water (Roche, UK) and 2 µl of template DNA. PCR was run on a LightCycler 96 real-time PCR machine (Roche, UK) under the following cycling conditions: pre-incubation of 95 °C for 600 s, 40 cycles of 95 °C for 10 s, 62 °C for 10 s and 72 °C for 30 s followed by a melting analysis to determine specific amplification. Any potential human positive samples were confirmed using the Promega Plexor® HY Human DNA forensic detection kit (Promega, UK). This assay was performed following manufacturer’s protocol using a Stratagene MX3005P (Agilent Technologies, USA) real-time PCR machine and analysed using the Promega®Plexor Analysis software.

### Statistical analysis

For each blood-fed mosquito, several variables were recorded: its species, the night and location (transect point and indoors/outdoors) of its capture, Sella stage and source host species of its blood meal. Wind speed and rainfall were also recorded for each capture night. Logistic regression was used to test the association between source species of blood meal (bovine vs non-bovine, and, human vs non-human) and i) species of mosquito, ii) transect point and iii) indoors/outdoors capture. Potential interactions between the variables and wind speed and rainfall were also sought. The Akaike Information Criterion (AIC) was used to estimate the relative quality of statistical models generated using intrinsic versus extrinsic factors for describing the data on blood meal sources. Our previous work identified that the level of blood meal digestion increased significantly the further away from the blood source that mosquitoes were caught^26^. Therefore, we conducted an ordered logistic regression to ascertain whether the Sella stage (for either bovine-fed or human-fed mosquitoes) varied significantly across the transect. All data analyses were performed in STATA. P values < 0.05 were assumed statistically significant.

## Results

### Summary of collection

A total of 904 blood fed *Anopheles* mosquitoes were collected across the 22 trapping nights. Of these, 665 (73.6%) were identified as members of the *Anopheles gambiae* species complex with 519 identified as *An. coluzzii* (57.4%), 70 were *An. gambiae ss* (7.7%) and 32 were *An. melas* (3.5%). 44 (4.9%) were *An. gambiae sensu lato* but could not be identified past the species complex level (Table 1). In addition, 108 (11.9%) were *An. pharoensis*, 54 (6%) were *An. rufipes* and 77 (8.5%) samples could not be morphologically or genetically identified (Table 2).

**Table 2:** Summary of blood-fed mosquito species collected by transect point and collection location.

Species	Collection Location*	T1	T2	T3	T4	T5	T6	Total
*An. coluzzii*	Indoors	100	202	15	12	4	21	354
	Outdoors	110	21	25	5	3	1	165
	Total	210	223	40	17	7	22	519
*An. gambiae* s.s.	Indoors	12	8	0	2	1	2	25
	Outdoors	35	5	3	0	1	1	45
	Total	47	13	3	2	2	3	70
*An. pharoensis*	Indoors	1	4	1	1	1	2	10
	Outdoors	65	20	8	1	4	0	98
	Total	66	24	9	2	5	2	108
*An. rufipes*	Indoors	0	17	0	0	0	0	17
	Outdoors	20	9	6	1	0	1	37
	Total	20	26	6	1	0	1	54
*An. melas*	Indoors	0	17	1	1	1	0	20
	Outdoors	3	2	4	1	2	0	12
	Total	3	19	5	2	3	0	32
*An. gambiae* s.l.	Indoors	1	24	1	0	0	0	26
	Outdoors	2	3	12	1	0	0	18
	Total	3	27	13	1	0	0	44
Unknown	Indoors	7	26	1	3	0	0	37
	Outdoors	22	9	3	2	3	1	40
	Total	29	35	4	5	3	1	77

*Indoor collections were performed in uninhabited outbuildings for T1 and T2 and inhabited houses for T3-T6.

A similar number of blood-fed mosquitoes were obtained from indoor (n = 493, 55%) and outdoor (n = 411, 45%) collections. Of the 493 collected indoors, 413 were shown to have blood meal sources of bovine origin (84%), 20 human (4%), nine (2%) of mixed human/bovine origin and 51 (10%) unknown. For outdoor collections, 364 (89%) had bovine blood meal sources, 6 (1%) had human sources, 10 (2%) human/bovine mix and 31 (8%) unknown.

The HBI and Bovine Blood Index (BBI) for each species and collection location is shown in Table 3. *An. coluzzii* had an overall HBI of 6%, with indoor HBI of 8.5% significantly higher than outdoor HBI of 0.6% (Pearson χ^2^ = 12.4068, p < 0.001). This general trend was maintained across the different species: overall, mosquitoes caught indoors had a higher HBI (Pearson χ^2^ = 8.7848, p = 0.003) and lower BBI (Pearson χ^2^ = 9.0013, p = 0.003).

**Table 3:** Summary of HBI and BBI calculated for each mosquito species and collection location.

Species	Collection location	Human Fed (HBI)*	Bovine Fed (BBI)*	Unknown (%)
*An. coluzzii*	Indoors (n = 354)	30 (8.47)	308 (87.01)	23 (6.50)
	Outdoors (n = 165)	1 (0.61)	154 (93.33)	10 (6.06)
	Both (n = 519)	31 (5.97)	462 (89.02)	33 (6.36)
*An. gambiae s.s*.	Indoors(n = 25)	2 (8)	22 (88)	3 (12)
	Outdoors (n = 45)	3 (6.66)	40 (88.89)	4 (8.89)
	Both (n = 70)	5 (7.14)	62 (88.57)	7 (10)
*An. pharoensis*	Indoors (n = 10)	2 (20)	8 (80)	1 (10)
	Outdoors (n = 98)	6 (6.12)	95 (96.94)	3 (3.06)
	Both (n = 108)	8 (7.41)	103 (95.37)	4 (3.70)
*An. rufipes*	Indoors (n = 17)	0 (0)	11 (64.71)	6 (35.29)
	Outdoors (n = 37)	1 (2.70)	32 (86.49)	5 (13.51)
	Both (n = 54)	1 (1.85)	43 (79.63)	11 (10.37)

*Includes mixed feeds.

### Impact of local host availability on mosquito host choice

Categorising all blood-fed mosquitoes as human-fed (n = 45) or non-human-fed (n = 859), logistic regression demonstrated a significant impact of the distance (in metres) from the village centre where a mosquito was caught (OR: 0.9790 (95% CI: 0.9749–0.9830), p < 0.001). This equates to approximately halved odds of being human-fed for mosquitoes caught 25 m from the village centre. Keeping human-fed mosquitoes as the response variable, logistic regression was repeated – first using mosquito (sibling) species as the (intrinsic) explanatory variable, then using transect point and trapping location (extrinsic) explanatory variables. Estimates of the Akaike information criterion identified the superiority of the model including extrinsic factors (model with extrinsic factors AIC: 243.0, versus, model with intrinsic factors AIC: 359.8).

Rainfall (yes/no) was not found to have a significant impact on whether or not a blood-fed mosquito sourced its meal from humans (p = 0.484) nor did it significantly modify the impact of indoors versus outdoors captures (p = 0.115). Windy nights (defined as nights with wind speeds recorded over 3 m/s) were also shown not to have a significant impact (p = 0.432) on whether or not a blood-fed mosquito sourced the blood meal from humans. However, windy nights were a significant modifier for whether human-fed mosquitoes were caught indoors versus outdoors (p = 0.041).

For mosquitoes which were bovine-fed (n = 796) or not bovine-fed (n = 108), a significant impact of the distance (in metres) from the cattle holding pen was found (OR: 0.9894 (95% CI: 0.9866–0.9922), p < 0.001). This equates to approximately halved odds of being bovine-fed for mosquitoes caught 50 m from the cattle pen which is an equivalent order of magnitude in difference found when comparing indoor- versus outdoor-caught mosquitoes (OR: 0.5875 (95% CI: 0.3644–0.9472), p = 0.029). Keeping bovine-fed mosquitoes as the response variable, logistic regression was repeated – first using mosquito (sibling) species as the (intrinsic) explanatory variable, then using transect point and trapping location (extrinsic) explanatory variables. Estimates of the Akaike information criterion identified the superiority of the model including extrinsic factors (model with extrinsic factors AIC: 611.3, versus, model with intrinsic factor AIC: 660.6).

Both rainfall and windy nights were negatively associated with mosquitoes having sourced their meals from cattle (respectively, OR: 0.2357 (95% CI: 0.1194–0.4654), p < 0.001; OR: 0.4830 (95% CI: 0.2902–0.8037), p = 0.005). Neither rainfall nor windy nights were a significant modifier for whether bovine-fed mosquitoes were caught indoors versus outdoors (respectively, p = 0.999 and p = 0.292). Ordered logistic regression did not find any significant association between the Sella stage of bovine blood meal digestion and distance from the cattle pen, nor between the Sella stage of human blood meal digestion and distance from the village centre (respectively, p = 0.081 and p = 0.464).

## Discussion

Mosquito vectors of human diseases differ markedly in their preference for human hosts^21,25,33^. This preference is driven by both intrinsic (genetic) and extrinsic (environmental) factors, meaning that the same species of vector may exhibit different host-biting behaviours when confronted with a different setting. Malaria vector species are often described as primary or secondary vectors and this is largely contingent on how much they typically bite humans. However, just how much flexibility a vector exhibits according to its local environment is poorly understood. A recent systematic review indicated that where a mosquito is caught (indoors or outdoors) may be as influential on host selection as which vector taxa is collected^23^. In a malaria endemic setting of southern Ghana, we sought to quantify the relative influence of extrinsic and intrinsic factors in determining which host species is bitten by local vectors.

Over several weeks during the rainy season, mosquitoes were collected across a range of alternative host (bovine or human) availabilities, both from aspirating indoors and from traps placed outdoors. A general preference for bovine blood hosts was demonstrated by local malaria vectors including sibling species that are considered anthropophagic (*An. gambiae* s.s. and *An. coluzzii*). Although atypical, the very low HBI for these vectors (respectively 7% and 6%) is not without precedent; a recent systematic review presented a very wide range of HBI values (~0–100%) found among *An. gambiae* caught across Africa^23^. More remarkable was the significant associations found between feeding indices and the extrinsic factors under investigation.

Across the different species found locally, blood-fed mosquitoes caught indoors had a significantly higher HBI and significantly lower BBI than those caught outdoors. Despite being an intuitive result, the number of studies describing both indoor and outdoor blood indices are quite limited; and most of these have sought to document the widely recognised generalist (‘catholic’) biting behaviour of *An. arabiensis*^34,35^. Perhaps, therefore, even the paragons of anthropophagy such as *An. gambiae* s.s. are just as unfussy in their host choice; but, we acknowledge that the result we have found in our field site would need replication elsewhere to develop the evidence base to confirm this speculation.

Regression analysis showed that whether or not a blood-fed mosquito obtained its meal from humans was better predicted by its proximity to humans and whether it was caught indoors than by the species of the mosquito. Similarly, whether or not a blood-fed mosquito obtained its meal from cattle was better predicted by its proximity to cattle and whether it was caught indoors than by the species of the mosquito. Sella staging of the blood-fed mosquitoes showed no significant difference in blood meal digestion across the transect. Hence, mosquitoes were moving freely over the 250 m transect indicating that there was no unobserved barrier between village- and cattle pen-adjacent mosquitoes. In other words, individuals from the same sibling species across the transect likely comprised the same mosquito population and observed differences in behaviour were indeed driven through extrinsic factors. We believe this to be the first evidence from the field demonstrating the high degree of potency that the relative influence of extrinsic factors has on host selection by a major disease vector. Our findings suggest categorizing biting behaviour solely on taxonomy or on a broadly spatial level (by region or country) risks missing the highly localized impact extrinsic factors such as host availability can have on mosquito host choice; and this could have significant public health consequences.

Malaria control is chiefly centered on mosquito control, and mosquito control is largely dependent on exploiting human-biting mosquito behaviour. The two most prominent malaria-control mainstays over the past 20 years in Africa have been insecticidal bednets, which target human-seeking mosquitoes, and indoor residual spray of insecticides, which targets mosquitoes that have recently fed. Historically, effectiveness of new formulations and combinations was assessed solely through measuring epidemiological endpoints (usually malaria incidence). More recently, clustered randomised trials have also incorporated the measurement of entomological endpoints^36–38^, following recognition that this is critical in linking insecticidal deployments with the mechanism(s) of disease control. However, monitoring of biting behaviour which fails to account for extrinsically driven plasticity risks misattribution of the mechanism(s) of disease control which would lead to misleading effectiveness measures and consequently jeopardise the rigour of any extrapolations or projections.

In addition to elucidating the mechanisms of effectiveness of current control trials and programmes, a better understanding of plasticity in host choice could qualitatively change disease control strategy. One burgeoning approach to malaria control is the treatment of blood-hosts with systemic insecticides. These insecticides are delivered topically, orally or parenterally and operate through the uptake of toxic compounds during a bite. They have been demonstrated in trials to significantly reduce malaria incidence^39^. These drugs are not restricted to human use and several studies have documented their efficacy in controlling mosquito vectors when applied to local livestock^40,41^. Models have demonstrated when and where this technology could be most successful either as a standalone tool^42,43^ or as part of an integrated vector management programme^44,45^. For these projection efforts to be built upon to inform operational control, reliable data are required for describing: how bites are distributed among different host species; how host choice is impacted by local host availability; and, how this behaviour is impacted in the presence of different control measures (e.g. bednets). In the setting of the present study, bednets were seen and used sporadically but also completely absent from some households during sampling. Therefore the host selection plasticity identified among the local vectors here could be exploited by complementing mass bednet deployments (i.e. a time that is arguably when local vectors will try to exploit alternative blood host species) with systemic insecticidal applications in livestock.

Conducting experiments such as those described in the current study but in different epidemiological/entomological settings – including communities before/after mass distribution of LLINs – is an important focus of future work. There is no reason to believe that host choice is always dominated by extrinsic factors – it is important to know how much this behaviour varies in different settings and what are the key drivers. Hopefully the ease with which the experiment is set up; its relatively modest costs; and, the broadly recognised criticality of human biting rate in malaria epidemiology will encourage others to capitalise on our methods to investigate mosquito biting behaviour in other settings. To that end, findings from this first study could be exploited by others in conducting power analyses to determine appropriate sample sizes for their mosquito collections.

## Conclusions

Local host availability is a powerful driver for host selection of even the most discerningly anthropophilic malaria vectors. The importance of not characterizing biting behaviour on a taxonomic level but through explicit study of local mosquito populations is demonstrated. A better understanding of plasticity in host choice is critical for attributing disease reductions to the correct control mechanisms and is key to implementing the most effective malaria control strategy.

## Data Availability

All data generated or analysed during this study are included in this published article.
